# Challenges and Strategies in Developing an Enzymatic Wearable Sweat Glucose Biosensor as a Practical Point-Of-Care Monitoring Tool for Type II Diabetes

**DOI:** 10.3390/nano12020221

**Published:** 2022-01-10

**Authors:** Sook Mei Khor, Joonhwa Choi, Phillip Won, Seung Hwan Ko

**Affiliations:** 1 Department of Mechanical Engineering, Seoul National University, 1 Gwanak-ro, Gwanak-gu, Seoul 08826, Korea; naomikhor@um.edu.my (S.M.K.); pass@snu.ac.kr (J.C.); 2 Department of Chemistry, Faculty of Science, Universiti Malaya, Kuala Lumpur 50603, Malaysia; 3 Department of Mechanical Engineering, Carnegie Mellon University, Pittsburgh, PA 15213, USA; philipwo@andrew.cmu.edu; 4 Institute of Advanced Machines and Design/Institute of Engineering Research, Seoul National University, Seoul 08826, Korea

**Keywords:** flexible patch, real-time sweat monitoring, type II diabetes, enzymatic and non-enzymatic wearable glucose biosensor

## Abstract

Recently, several studies have been conducted on wearable biosensors. Despite being skin-adhesive and mountable diagnostic devices, flexible biosensor patches cannot truly be considered wearable biosensors if they need to be connected to external instruments/processors to provide meaningful data/readings. A realistic and usable wearable biosensor should be self-contained, with a fully integrated device framework carefully designed and configured to provide reliable and intelligent diagnostics. There are several major challenges to achieving continuous sweat monitoring in real time for the systematic and effective management of type II diabetes (e.g., prevention, screening, monitoring, and treatment) through wearable sweat glucose biosensors. Consequently, further in-depth research regarding the exact interrelationship between active or passive sweat glucose and blood glucose is required to assess the applicability of wearable glucose biosensors in functional health monitoring. This review provides some useful insights that can enable effective critical studies of these unresolved issues. In this review, we first classify wearable glucose biosensors based on their signal transduction, their respective challenges, and the advanced strategies required to overcome them. Subsequently, the challenges and limitations of enzymatic and non-enzymatic wearable glucose biosensors are discussed and compared. Ten basic criteria to be considered and fulfilled in the development of a suitable, workable, and wearable sweat-based glucose biosensor are listed, based on scientific reports from the last five years. We conclude with our outlook for the controllable, well-defined, and non-invasive monitoring of epidermal glucose for maximum diagnostic potential in the effective management of type II diabetes.

## 1. Introduction

**Figure 1:**
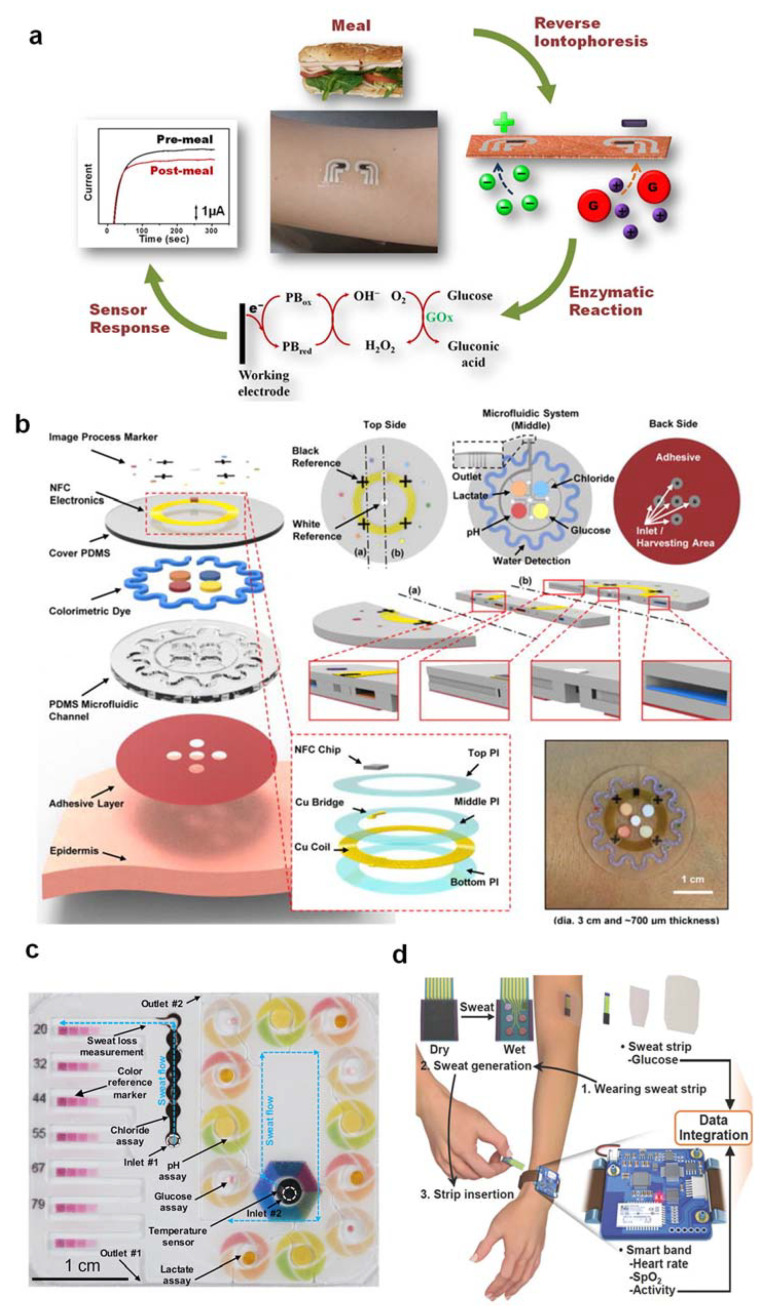
(**a**) A tattoo-based glucose-sensing platform that is non-invasive. (**b**) Schematic illustrations, optical images, and theoretical stress modeling of an epidermal microfluidic biosensor for sweat monitoring integrated with flexible electronics. (**c**) Soft, flexible microfluidic devices for colorimetric sweat analysis on the skin. (**d**) Schematic diagram of a wearable health-monitoring device consisting of a disposable sweat test strip for measuring glucose and a wearable smart band for monitoring heart rate, blood oxygen saturation, and physical activity. The figures are reprinted with permission from ref [3,4,5,10].

According to the 10 November 2021 report of the WHO, diabetes mellitus was the cause of 1.5 million deaths in 2019. Nearly 50% of all deaths were attributable to the presence of high blood sugar before the age of 70. The WHO estimates that diabetes was the ninth leading cause of death in 2019 (WHO, 2021) [1] (“WHO. Diabetes”. http://www.who.int/mediacentre/factsheets/fs312/en/, accessed on 10 November 2021). Diabetes can be avoided or treated with effective and consistent physical exercise, a low carbohydrate/sugar diet, proper medication, and, most importantly, continuous monitoring and timely care to avoid complications. Despite this, mortality rates related to diabetes are rapidly increasing. A medical diagnosis of blood glucose or HbA1c monitoring is not only costly but also causes pain and stress in addition to the risk of potential infection. Non-invasive wearable glucose biosensors for sweat, saliva, and tears have been developed to overcome these obstacles. Owing to their simplicity, sweat-based biosensors have received relatively high attention. Over the years, sweat-related physiological work has been well-founded. Olarte et al. (2013) used a digital nose to detect glucose in human sweat [2]. However, off-body sweat glucose biosensing is tedious, as sweat samples must be collected, stored, and tested remotely in a laboratory by trained professionals using costly and bulky equipment. In addition, difficulties in obtaining adequate sweat sample volumes (≥10 μL), evaporation, and chemical degradation between sweat collection and testing have limited the sensitivity and reliability of these off-body tests. Consequently, the real-time, accurate, and routine monitoring of people’s diabetic health status has been compromised. Thus, wearable sweat glucose biosensors that enable the collection and analysis of sweat in real time, and an autonomous, integrated wearable system enabling reliable, continuous, and painless monitoring of glucose with minimal intervention by specialists have become a requirement. It took only about five years to explore wearable sweat-based glucose-biosensing devices [3,4] (Figure 1a,b). For realistic point-of-care monitoring, it is necessary to fully understand sweat dynamics and explore wearable glucose biosensors. These should be stable and reliable for a long time (≥24 h) and be capable of assessing sweat glucose in real time, thereby accurately indicating the actual physiological condition of the human body. In addition, for continuous-glucose-monitoring purposes, certain easily accessible sweat-collection sites containing eccrine glands (sweat glands known to secrete sweat-containing glucose) such as the forehead, forearm, and back should be selected [5]. Glucose is one of the major sweat-secreted metabolites that are at a much lower concentration in sweat than in the blood. Specifically, glucose concentrations range from 2 to 40 × 10^−3^ M in the blood, whereas in sweat they are in concentrations of only 0.01–1.11 × 10^−3^ M [6]. Low concentrations (~100 times dilution) of glucose in sweat require highly sensitive systems, particularly in the case of hypoglycemia or skin glucose residue contamination, which cause strikingly high biases in sweat glucose. A majority of the wearable sweat glucose biosensors recently reported are either colorimetric or electrochemical dependent. As a point-of-care diagnostic tool for better management of type II diabetes, colorimetric-based glucose biosensors generally have lower operating costs compared to electrochemical-based glucose biosensors (a fully integrated device is expensive). Accurate, real-time, and reliable concentration readings of sweat glucose (after auto corrections for sweat rate, pH, and temperature) can only be obtained using electrochemical biosensors. Both methods are suitable for long-term continuous glucose monitoring, but the disposable biosensing patch needs to be replaced after each measurement, especially for colorimetric biosensors. The cost of the materials used and the bio/chemical reagents used to develop a wearable glucose biosensor are, therefore, crucial. To be a viable diagnostic tool, the system/patch should not be priced higher than any of the current commercially available personal blood glucose meters; otherwise, despite being non-invasive, the technology cannot be considered truly beneficial for the effective management of type II diabetes. In this review, we also address the limitations of each strategy and the advanced approaches identified to solve the respective problems.

### 1.1. Wearable Sweat Biosensors Based on Colorimetric

A powerless operating system and simple RGB color signal interpretation have established colorimetric biosensing as an eminent method for sweat glucose analysis. However, colorimetric biosensing works only for enzymatic glucose biosensors, as non-enzymatic glucose biosensors require electron transmission (glucose oxidation) and, therefore, only work in electrochemical sensing.

Digital-imaging techniques are used in colorimetric chemical tests to extract quantitative information. Despite impressive progress, some inadequacies have been found, particularly about accurate, simultaneous, and multianalyte analyses across physiologically relevant concentration ranges. These deficiencies are due to poor design. Continuous sweat streams through the enzyme reaction zones can cause chemical diffusion and, therefore, color leaching. Time-dependent and spatially non-uniform color responses limit the precision and reliability of the measurement [7]. Errors associated with irregular color production on filter paper are also caused by uncontrollable capillary wrapping through the paper and concentrated edge effects [8]. Isolated color reference markers may be mounted in the peripheral regions of the wearable sensing system; however, they cannot ensure accurate color analysis, owing to inconsistent lighting conditions [9]. Furthermore, in the case of colorimetric sweat glucose analysis, it is necessary to avoid any overlap between sweat-sampling processes and glucose-biosensing processes, as colorimetric reagents and reaction products could cause severe skin burns (e.g., o-Dianisidine).

Several successful attempts towards practical demonstrations of microfluidic platforms for accurate and multiplex colorimetric analysis of glucose, lactate, pH, chloride, and sweat in a wide variety of ambient lighting conditions have been reported [4]. (Figure 1b). To summarize, white and black reference color markers were implemented to reduce reliance on the lighting conditions of realistic significance (daylight, shadow, and various light sources). A white dot was mounted in the middle of the unit, and four black crosses were symmetrically distributed near the center to calculate values for 100% and 0% of the RGB coordinates, respectively. Precise analyses of sweat rate and sweat volume in the microfluidic serpentine channel are performed by referring to the black crosses even when the images are rotated or translated. The digital color data (in RGB format percentage) were then converted to real analyte concentrations after image correction using a calibration chart. A thin, skin-mountable microfluidic interface platform with multimodal sweat glucose-biosensing capabilities was developed to overcome the problems caused by filter paper. A collection of optimized capillary-bursting valves in the device was used to direct the sweat flow to individual micro-reservoirs for separate, multiple test reactions within a single device, preventing cross-contamination or flow-through mixing effects. In the liquid phase, the color design was carried out to resolve any uneven color development and allow spatially uniform adjustments for accurate homogeneous color measurement [4] (Figure 1b). Multiple color reference markers are printed directly on the surface of the biosensing platforms, adjacent to each of the micro-reservoirs, to enable real-time quantitative analysis under various lighting conditions [10] (Figure 1c). Chemical assays must be designed to accurately measure the concentration of sweat biomarkers across physiologically relevant ranges. The color checker can be used to correct images taken with ambient lighting to achieve accurate results similar to those obtained under controlled lighting conditions.

### 1.2. Wearable Sweat Biosensors Based on Electrochemical

Recent developments in skin-integrated electronic platforms support the analysis of sweat glucose and wireless data-transmission hardware using electrochemical biosensors. While these types of wearable biosensors allow continuous monitoring of sweat glucose levels, they usually require batteries in addition to other supporting electronics (wireless PCBs) and subsystems that can dominate the form factor [11]. The electrochemical sweat glucose biosensors could be manufactured in a two-electrode configuration on flexible elastomer substrates, a common strategy for low current consumption in electrochemical biosensors. In some situations, the airtight cover layer used, which is typically waterproof to avoid sweat evaporation, results in an irritable wearing experience [12], especially when the patch is intended for long-term skin adhesion to enable continuous sweat glucose monitoring. To overcome this issue and increase functional applicability, the patch model should be either disposable or reusable, that is, it should be attachable multiple times [5] (Figure 1d).

## 2. Wearable Biosensors for Non-Invasive Control of Sweat Glucose

Two major types of wearable glucose biosensors, enzymatic and non-enzymatic sensors, have been identified to date. Enzymatic biosensors allow for both colorimetric and electrochemical biosensing. Non-enzymatic sensors, on the other hand, are only suitable for electrochemical sensing because glucose detection requires redox reactions. On account of the selectivity and stability of commercial biosensor strips, all commercially available blood glucose meters are enzymatic glucose biosensors [13]. Non-enzymatic systems, on the other hand, neglect electrode selectivity, slow glucose oxidation kinetics in many unmodified electrodes, electrode fouling by biological fluid sample constituents, and a limited number of physiologically relevant pH systems [14]. Therefore, non-enzymatic glucose sensors would not meet strict requirements to be commercially viable, thus ruling out a wearable non-enzymatic glucose sensor. In other words, wearable enzymatic sweat glucose biosensors have exciting potential as a point-of-care diagnostic tool for better management of type II diabetes.

### 2.1. Enzymatic Biosensors

The use of enzymes, however, results in several critical problems about wearable glucose biosensors. First, both wearable electrochemical and optical glucose biosensors based on the enzyme glucose oxidase (GOx) are highly sensitive to changes in the sweat environment, such as temperature, pH, and ionic strength, which cannot be regulated within in situ conditions [15]. Prolonged sweat pH is typically lower (pH 4.5–6) than neutral physiological pH (pH 7), owing to metabolic lactic acid production during muscle movement; furthermore, prolonged sweat pH varies between humans. After denaturation, enzymes cannot recover their characteristics owing to acidic pH, high temperature, or high ionic strength. Second, in addition to enzyme degradation over time, another concern is poor stability. Even in the case of highly glycated and therefore stable GOx, its catalytic activity will decrease slowly with time, affecting its shelf life and long-term wearable monitoring capability (increase in skin temperature during exercise, normal body temperature: 36.5–37.5 °C) [5]. Third, on exposure to frequent mechanical friction and skin deformation, delamination of the immobilized enzyme layer from the biosensor interface may occur. These limitations decrease the long-term storage stability and the reliable continuous usability of the sensors in real-time monitoring. Fourth, the immobilization of enzymes on the transducer surface involves processes such as covalent attachment, cross-linking polymerization, or sol-gel entrapment on the surface of the working electrode. This not only suppresses the activity of the enzyme but also immobilizes electrode reagents, thereby slowing down the transmission of electrons and reducing the sensitivity of detection. Fifth, commercially available enzymes have a high cost and are generally bio-sourced agents suitable for in vitro analyses only, and therefore may not be appropriate for wearable biosensing. Sixth, wearable sweat-based biosensors, with a closed air gap to prevent sweat evaporation, have only a two-phase solid–liquid interface whereby oxygen is transmitted through the liquid phase (excreted sweat) with a low diffusion coefficient as compared to oxygen in the air. Consequently, the supply of oxygen available in the enzyme reaction zone is limited by Fick’s law; this restricts the upper limit of linearity, sensitivity, and accuracy detection.

**Figure 2:**
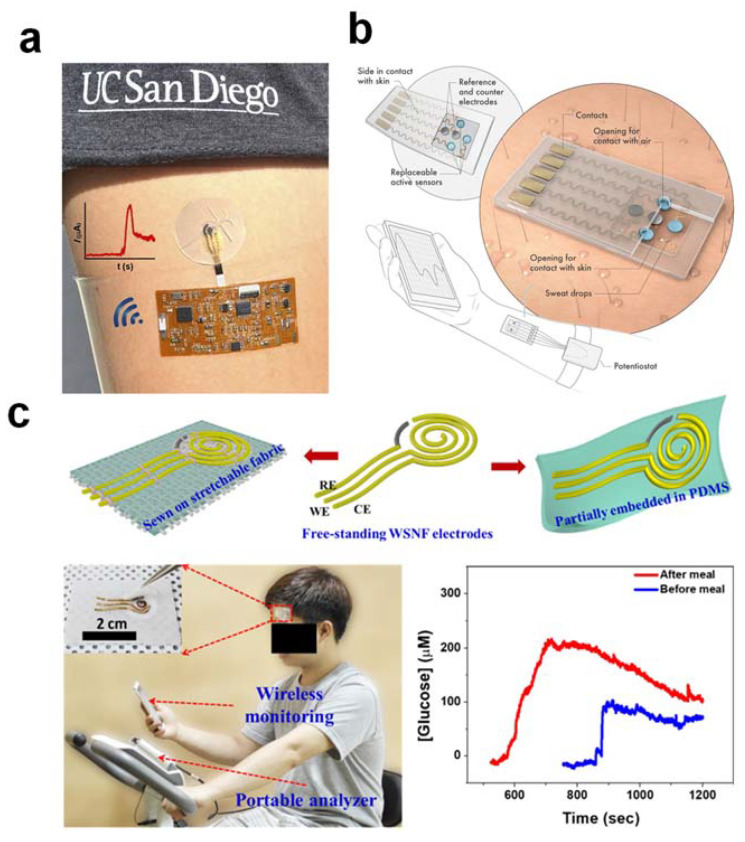
(**a**) Epidermal electrochemical detection system integrated with conformal wireless electronics on the skin. (**b**) A wearable biosensor system based on MXene (a three-phase oxygen-rich electrode design) is depicted schematically. (**c**) A wrinkled, stretchable, nanohybridfiber glucose sensor for continuous on-body monitoring before and after meals of glucose levels in sweat. The figures are reprinted with permission from ref [17,18,21].

To overcome substantial temperature changes, continuous monitoring of sweat glucose is ideally carried out indoors at a constant room temperature. The body temperature at 37 °C would remain stable at rest by using iontophoresis to generate sweat, making it suitable for optimal enzyme activity to detect glucose. Integration of a flexible microfluidic system with a wearable glucose biosensor will allow the instantaneous flow of old acidic sweat through the biosensing electrode and immediately replenish it with new, fresh sweat at a near-neutral pH, thereby overcoming the problem of acidic sweat pH due to the excretion of lactic acid. Due to the short contact time between acidic sweat and immobilized GOx enzyme, the enzyme would not be degraded unless it was constantly immersed in acidic sweat in the absence of a microfluidic system. Long-term enzyme stability in contact with human skin can be achieved by designing a disposable type of sweat glucose biosensor with the frugal chemical sensing strip replaced for each measurement, but not for the whole integrated system. It is necessary to design a flexible type of glucose-biosensing electrode to overcome the problem of enzyme delamination, but the electrode itself must be truly stretchable. In addition, a thin, soft, and flexible outer cover layer to avoid sweat evaporation will help protect the enzyme and prevent delamination from the biosensing interface. Incorporating advanced nanomaterials (e.g., MOF, polymer brush) into the biosensing electrode interface and biological/chemical modification of enzymes [16] could significantly improve the rate of electron transfer and sensitivity to glucose sensing, thus resolving enzyme immobilization problems. The multilayer biosensor model should hold the immobilized enzyme at a minimum distance from the skin to avoid near-direct contact with the wet skin surface and to undermine the potential for skin irritation or any adverse effects due to the use of bio-sourced enzyme grade in a wearable glucose biosensor [17] (Figure 2a). To address the problem of low oxygen supply, Lei and co-workers have developed a new wearable sweat-based biosensor with an open-air hole and a three-phase reaction interface, thus providing an ample and constant supply of oxygen [18] (Figure 2b). To realize this concept, the outer, isolating cover layer is made of soft silicone rubber with air holes deliberately introduced. This allows oxygen to diffuse easily from the air to the active biosensing interface. As the oxygen diffusion coefficient in the air phase (2.0 × 10^−1^ cm^2^ s^−1^) is much higher than that in the sweat solution phase (2.1 × 10^−5^ cm^2^ s^−1^) [19], the oxygen level in the tri-phase reaction zone remains relatively constant and can be higher by several orders of magnitude. An oxygen-rich enzyme electrode ensures rapid oxidation of glucose and the formation of an abundant amount of H_2_O_2_, which in turn strengthens the signal, allowing more accurate and consistent testing, a wider linear detection range, and ultra-high glucose sensitivity.

### 2.2. Non-Enzymatic Sensors

The development of a wearable sensor for non-enzymatic electrochemical detection of sweat glucose could be an alternative, given the challenges and limitations faced by enzymatic biosensors. The detection of glucose using non-enzymatic electrochemical sensors has been studied for a long time [14]. Electrocatalytic materials capable of oxidizing glucose quickly and effectively (including bulk metal, metal oxide, alloys, metal nanomaterials, and carbon nanocomposites) are used in these sensors [20]. One of the most critical drawbacks for wearable sensing is that electrocatalytic glucose oxidation must be carried out in an alkaline environment (pH > 11) for appropriate selectivity, sensitivity, and reproducibility. Conversely, a drastic reduction in or even complete loss of electrochemical reactions is observed if the analysis is made at the physiological pH of sweat (pH 7.2–7.3). Glucose detection under non-alkaline physiological conditions shows poor reproducibility results due to severe electrode-surface poisoning as competitive chloride adsorption (Cl^−^) and phosphates (PO_4_^3−^) passivate the sensing interface, resulting in unsatisfactory reliability of sensing. Non-enzymatic transition-metal sensors have a linear range inappropriate for the diagnosis of blood glucose (2–20 mM) or sweat glucose (sweat glucose level: 0.2–0.6 mM). Non-enzymatic sensors using metals or alloys, such as Pt and Pt-Pb alloys, are known to be expensive, toxic, and have poor selectivity [20].

Zhu and co-workers have recently developed a non-enzymatic wearable sensor for electrochemical analysis of sweat glucose under alkaline conditions. This is to resolve the restricted working pH environment, specifically at alkaline pH for non-enzymatic wearable sweat glucose sensors [11]. A new strategy was used in their research by applying multi-potential steps to a non-enzymatic gold sensing electrode. A high potential negative step (−2.0 V) was first used to pre-treat the sweat sample, producing a localized alkaline condition on the electrode surface. Following this, a moderate sweat glucose-detection potential (0.2 V) was applied under the alkaline condition as generated in the first step. In the final step, a positive potential (1.0 V) was applied to the electrode surface for cleaning and regeneration purposes. Due to its high electrocatalytic activity towards glucose oxidation, Au was used as the working electrode in an alkaline condition. However, the multi-potential step approach causing pH change was only located within the diffusion layer near the wearable electrode-sensing interface, much smaller in volume than the bulk solution. Therefore, during the glucose-sensing process, it did not cause skin irritation [11].

Alternatively, for sweat glucose detection under neutral conditions, Toi et al. (2019) have developed a new electrochemical patch design consisting of a wrinkled, stretchable, and nanohybrid fiber with a high overall surface area [21]. This can provide a high electrocatalytic effect, stretchability, and durability under mechanical deformation. Synergetic effects between the Au nanowrinkles and the rGO supporting matrix, with oxygen-containing functional groups, improved the electrocatalytic behavior of the sensor by inducing abundant hydroxide anions on the Au nanowrinkled surface, facilitating dehydrogenation in the glucose oxidation reaction. This innovative technique can be used to detect glucose under neutral pH conditions and is therefore ideal for wearable non-enzymatic sweat glucose sensing. In addition, the stretchable, non-enzymatic glucose sensor has a stretching capability of up to 30% and remains stable after 10,000 stretch cycles at a strain of 30%. This provides high mechanical durability under repeated mechanical deformation cycles [21] (Figure 2c).

For wearable non-enzymatic glucose sensing, to reduce interference from the extracted sweat sample matrix and subdue sensor selectivity problems, the electrode could be covered with a Nafion layer followed by another Kel-F membrane layer. The Nafion is a cation-exchange polymer membrane that can selectively exclude anions from the electrode surface [22]. A Kel-F membrane is a type of fluorocarbon material that can repel charged molecules (e.g., amino acids, acids, urea, ammonium) [23]. On the other hand, transition-metal oxides, such as NiO or Co_3_O_4_, can replace Pt and Pt-Pb alloys and show high sensitivity. Mixed transition metal tungstate has recently gained a great deal of attention due to its remarkable properties, occurring due to different valence states of the W atom. Furthermore, tungsten material has the advantages of being simple to synthesize, inexpensive, low in toxicity, and highly stable [20].

## 3. Ten Specific Requirements for the Development of a Desired Wearable Sweat-Based Glucose Biosensor for Successful Type II Diabetes Management: Challenges and Solutions

Nonetheless, recent studies on sweat glucose analysis indicate that while the sweat can be collected non-invasively on the skin’s surface for functional and real-life applications, many primary analytical challenges remain to be addressed before a successful commercial operation. This includes: (i) limited basic knowledge of sweat biology with uncertainties in real-time sweat glucose concentration due to varying sweat flow rates; (ii) biofluid association such as blood and interstitial fluid; (iii) biosensor reproducibility, individual biosensing consistency, and regular biosensing reliability across large population studies. To achieve a desirable, workable, and practical wearable glucose biosensor for effective management of type II diabetes, ten basic criteria must be met, ranging from device manufacturing, proof-of-concept, marketing, and real-world application. The challenges, limitations, and advanced strategies used to effectively address all problems encountered in the achievement of each criterion are discussed.

### 3.1. A Fully Integrated and Autonomous Platform

**Figure 3:**
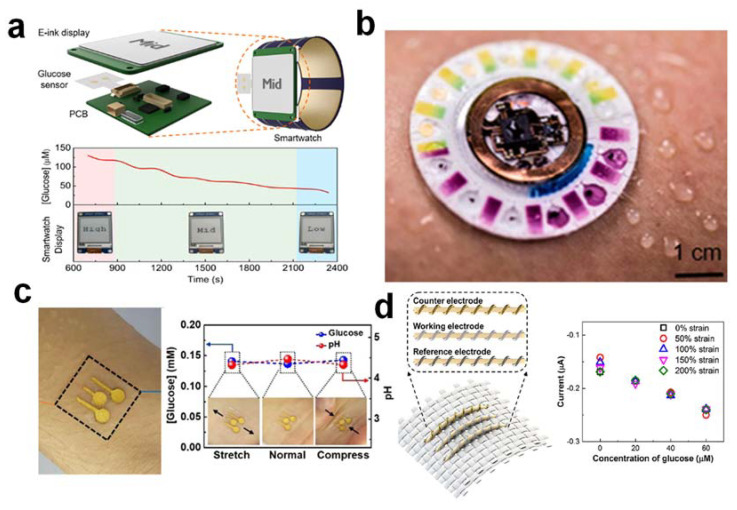
(**a**) It is a fully integrated, self-powered smartwatch for continuous sweat glucose monitoring. (**b**) It is a skin-interfaced, battery-free microfluidic system for electrochemical, colorimetric, and volumetric sweat analysis. (**c**) It is a skin-attachable and stretchable electrochemical sweat sensor for glucose and pH detection. (**d**) It is a fiber-based electrochemical wearable biosensor that is highly stretchable and strain-insensitive for monitoring sweat glucose. The figures are reprinted with permission from ref [25,28,29,31].

A self-contained wearable sweat glucose biosensor must be a fully integrated and autonomous system [24]. For non-invasive, real-time, and continuous monitoring of sweat glucose levels, the device should consist of a flexible photovoltaic/biofuel cell for energy harvesting and conversion, flexible and secure rechargeable batteries (e.g., Zn-MnO_2_) as intermediate energy storage devices, an iontophoretic system for autonomous on-demand sweat extraction (at least 100 nL/min/cm^2^) with a well-controlled sweat generation rate, a microfluidic system for sweat collection and temporary storage, an electrochemical/colorimetric biosensing platform with functional electrodes to convert concentrations of sweat glucose into electrical/optical signals, a flexible printed circuit board (PCB) to drive the iontophoretic process and to allow in situ data analysis as a controlling module (e.g., processing, calibration, and easy read-out signal transmission), and preferably a digital screen for direct and real-time tracking of glucose, removing transmitting systems and external reading devices. In addition, the electronic display module should ideally provide a stable display without requiring continuous power, ultra-low power consumption, a wide-viewing angle, high visibility, and good contrast. The sweat glucose level is indicated as “low”, “mid”, or “high”, with its respective corrected values depending on the sweat glucose concentration detected with pH or temperature changes taken into consideration during in situ measurement. This fully integrated system offers operational convenience, safety, and miniaturization that are highly desirable for wearable and portable applications. A miniaturized sensor design allows, normally, an infinitesimal amount of sweat for accurate sweat glucose analysis. Without additional data storage and download capabilities, however, this topology prevents data storage or dissemination to health care providers and is, therefore, less attractive for integrated health-monitoring applications [25] (Figure 3a). To overcome this limitation, most of the currently reported wearable glucose biosensors rely primarily on wireless data transmission (e.g., near-field communication (NFC), Wi-Fi, Bluetooth, and Zigbee) and external display components (e.g., mobile phones, computers) for real-time data analysis and feedback for practical self-monitoring applications. Continuous monitoring of sweat glucose for 24 h or longer is important. It is certainly commercially relevant to have this capability, with the only downside being the restricted miniaturization of the whole system.

Although blood temperature and acidity remain homeostatic, these parameters in sweat can differ with varied generation conditions, and this can weaken enzyme-detection reliability. The optimized sweat glucose biosensors should therefore be coupled with pH and temperature sensors in the integrated sensing system/patch for more precise calibration [10]. Enzyme activity in the biosensing layers can change with pH. During muscle movements, metabolic secretion of lactic acid in sweat decreases the pH level to 4–5. The pH value varies between subjects and is affected by dietary routines [26]. Consequently, a change in glucose biosensor signal can result due to a change in sweat pH instead of fluctuations in secreted glucose. This problem can be solved by parallel sensing of glucose and pH. Next, biosensor signals can also be influenced by ambient and skin temperature. The increase in temperature leads the glucose biosensor to overestimate the amount of sweat glucose, as higher temperatures increase enzymatic activity. Temperature and pH sensors integrated with glucose biosensors enhance the accuracy of glucose monitoring by providing real-time corrections of the measured sweat glucose levels based on pre-calibrated data. It is also important to test several sweat analytes at the same time as sweat glucose, as this can help decide if changes in biosensor signals are due to real changes in analyte concentrations or sweat rate effects. Potassium levels in sweat, for example, are quite invariant concerning sweat rates and normal physiological changes in the body. Therefore, if there is a change in the signals of sodium, lactate, or glucose while the potassium signal is constant, the other changes in the sensor can be confidently triggered by a real physiological event [4]. In other words, it is essential to integrate sweat-rate sensors (K^+^, Na^+^) to unravel the rate-dependent sweat-glucose-secretion modulation. By contrast, a single analyte biosensor could generate misleading information as a change in signal could be due to sweating stoppage, detachment of the biosensor from the skin surface, or even biosensor failure. Thus, multiple sensors can be used for accurate detection. High reproducibility with minor variations makes it possible to calibrate one-point biosensors before use. One-point calibration is required to account for baseline differences in biosensor signals, but a universal calibration curve ensures that only one preliminary measurement is required to minimize the complexity of the device setup and prepared before use on the body. Such wearable glucose biosensors should be fitted with a single calibration curve so that there is no need for extensive testing of the sensing characteristics of each unit individually before use [27]. This is standard practice for electrochemical biosensors in particular because even commercial pH meters need pre-calibration in generic buffer solutions.

A fully integrated and autonomous device should consist of a multilayer stack of at least three subsystems for wearable colorimetric sweat glucose biosensing: (i) an excellent skin-compatible adhesive layer (including wet skin) with microchannel openings identifying sweat-collection and sweat-rate-monitoring areas; (ii) a sealed array of thin, elastic microfluidic channels and multiple reservoirs filled with color-responding materials for different analyte concentrations; and (iii) near-field electronics (NFC) to communicate with existing wireless devices [28] (Figure 3b). The eccrine glands themselves will provide pressure to route sweat through a network of microfluidic channels. One-way control valves are used to communicate with the individual, separately located reservoirs containing color-responsive materials in a manner that avoids contamination and crosstalk. The main advantage of a fully integrated, wearable, colorimetric sweat glucose biosensor is the lack of a requirement for a power supply to run the whole system and analyze glucose. One of the limitations, however, is that it can only be used to analyze sweat glucose produced by physical exercise and not by iontophoresis or stretchable heaters. Therefore, this is not suitable for people who are sedentary.

### 3.2. A Wearable Material That Is Soft, Flexible, and Stretchable

When designing a glucose biosensor wearable for on-body monitoring, stretch-ability and structural stability (active biosensing electrode surface) are two significant competing issues requiring careful design and optimization. Although thin geometries and low-modulus elastomers are crucial to mechanical compatibility with the body, these characteristics often result in significant deformation or even fracturing by external pressure, either during fabrication or in conditions associated with natural skin deformation. Thus, the wearable sweat glucose biosensors should be fabricated with flexible, thin, and stretchable hybrid features so that they conform to the body surface and withstand physical strain [29] (Figure 3c). These unique features make it easy to wear and stable even when skin contact is physically disturbed. Mechanical friction and deformation of devices on soft human skin will delaminate the enzyme from the glucose biosensor. Materials and devices straining can complicate the signal performance of the sensor and degrade the reliability of the biosensor patch. Therefore, during normal activities of the wearer, it is important to mitigate movement-induced artifacts to avoid signal interference. The stretchability of wearable biosensors can be accomplished either by using internally stretchable electrodes or by using extrinsically stretchable structures. Extrinsic structural development can be achieved through the introduction of island-bridge, serpentine, wavy, or helix structures while intrinsic development can be achieved through the use of liquid metal nanomaterials, noble metal nanowires (Ag nanowires), gold nanomaterials (gold fiber), and carbon nanotubes (CNT) that may converge into percolation networks [29]. In addition, the wearable biosensor should be able to detect multi-directional strain to emulate the strain environment and ensure mechanical stability during repeated stretching cycles [21]. Multidimensional strain biosensors are difficult to fabricate as they usually show high coupled electrical conductivity changes in the main axis of the principal strain and perpendicular direction due to the Poisson’s ratio [30]. Under a large strain condition, this issue becomes more serious. Stretchable biosensors of sweat glucose could be fabricated using conventional techniques of evaporation and photolithography [29]. The disadvantage of these techniques is that they require a high vacuum, which is time-consuming and costly. The benefits of using the filtration system, on the other hand, are low cost, low temperature, simplicity, and easy process [29]. Specific deformation tests should be performed to determine the mechanical stability of the biosensor under mechanical stresses anticipated during operation on the body. The wearable device should be bent, twisted, and stretched when applied to the arm to test its resistance to such stresses and the presence of potential cracks or breaks in the electrode surface [4]. If these strains do not cause any apparent damage to the structure, it reflects the conformity and flexibility of the whole integrated biosensing system. The wearable biosensor should ideally be mechanically stable after 1000–10,000 repetitive stretching/releasing cycles at 50% strain [31].

### 3.3. Real-Time Sweat Stimulation and Extraction

Sweat is an emerging, non-invasive biofluid that can be readily accessed through physical exercises or deliberately generated on demand using low electrical current through thermal heating or chemical induction called iontophoresis. Significant advantages and numerous recent reports have focused on on-body sweat analysis [32]. Successful implementation of a reliable, non-invasive, and affordable monitoring system for the effective management of type II diabetes has many primary challenges and limitations related to real-time sweat stimulation and continuous and long-term sweat glucose monitoring. These challenges include: (1) irregular or low sweat-generation rates during exercise; (2) being unable to perform regular sweat-generating exercise due to health problems such as severe obesity, heart problems, or unfitness due to age (senior citizen), pregnancy, illness, or disability; (3) iontophoresis limitations that are only promising for one-time measurement; and (4) an uncomfortable and unbreathable wearing experience induced by the stretchable wearable heater. Meeting these challenges requires substantial improvement in sweat induction, storage, and transportation.

#### 3.3.1. Physical Exercise

For effective treatment of type II diabetes, glucose levels for diabetic patients are well controlled by periodic insulin injections before or during meals or routine prescription of drugs. A continuous, reliable, non-invasive attempt to monitor sweat glucose levels is therefore very important to assess the appropriateness of care provided and to minimize morbidity and mortality related to complications caused by diabetes. One of the options could be physical exercise. However, diabetic patients require sweat glucose continuous monitoring six times a day (fasting before breakfast, after breakfast, lunch, after tea, dinner, and before sleep), and showering six times a day due to an uncomfortable feeling after excessive sweating is not practical from a private behavioral standpoint. Even a healthy, non-diabetic subject is also unable to endure six-fold physical exercise to generate sufficient sweat for several continuous sweat glucose-monitoring sessions per day. No diabetic patient is willing to do physical exercise every day to generate sweat for non-invasive sweat glucose monitoring and maintain it for many months or years to effectively manage and control diabetes. Intensive exercise to produce sweat glucose can also cause hypoglycemia in diabetic patients, and this is one of the major concerns of diabetic patients. Although a non-invasive approach to glucose control, the sweat generation process by physical exercise is not completely functional. Diabetic patients with severe obesity or pregnant women may reach their maximum oxygen intake earlier, resulting in a tendency to sweat earlier but showing lower sweat rates compared to normal diabetic patients. On the other hand, as perspiration begins, glucose levels in sweat gradually decrease with time as glucose is rapidly flushed out of the sweat gland. The decreased levels of sweat glucose are attributed to the dilution effect caused by an increase in sweat rate, which is observed visually as exercise continues. As exercise occurs, apart from the rise in the sweat rate causing glucose dilution, the increase in skin temperature will also affect the activity of the enzyme (GOx), which must be accounted for to avoid overestimation of the actual concentration of glucose. However, different body parts show different sweat rates, which, despite similar trends, results in different concentrations of glucose at any particular time due to the dilution effect. To decipher sweat glucose more holistically, comparing glucose variations across body locations, environmental parameters, and reactions to glucose intake would be more helpful. In short, real-time, continuous, and accurate sweat glucose monitoring achieved through exercise sweat is highly unlikely to be an ideal approach, particularly for patients who have diabetes in addition to other serious health problems such as obesity and cardiovascular disease. Additionally, this approach is unlikely to be ideal for pregnant women, the elderly, and the disabled.

#### 3.3.2. Iontophoresis

**Figure 4:**
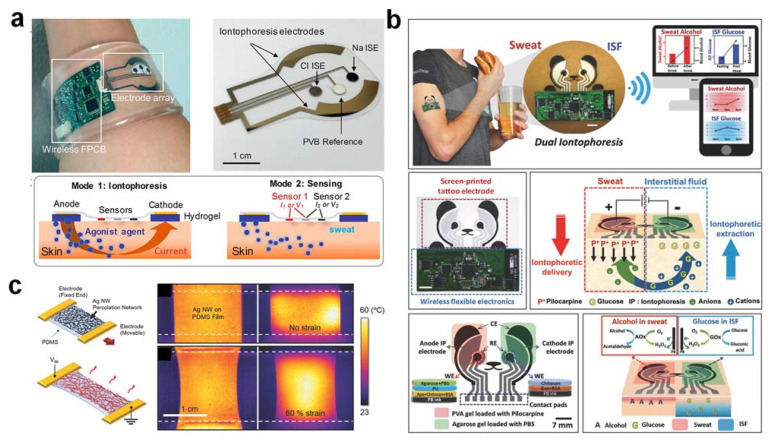
(**a**) A fully integrated wearable sweat-extraction and analysis platform for cystic fibrosis and glucose monitoring. (**b**) Monitoring of sweat (iontophoresis) and interstitial fluid (reverse iontophoresis) with a single wearable biosensor system at the same time. (**c**) A highly stretchable and transparent nanowire heater for wearable electronics applications. The figures are reprinted with permission from ref [12,33,35].

Iontophoresis is an established technique used to polarize ions and polar molecules at each electrode by applying a mild electrical current throughout the skin and is commonly used in clinics for diagnostic and therapeutic purposes, especially for sedentary people. A sweat-inducing agent such as pilocarpine (stimulating agonist) is iontophorized into the skin (sweat glands) to generate sweat in the iontophoresis process [3]. Hence, at any convenient location on the body, sweat can be produced without invasion and on-demand. Embedded iontophoresis capabilities should be included in a well-integrated system for local sweat extraction. Using a pair of ring-shaped electrodes (WR Medical Electronics Co., area: 4.3 cm^2^), with the sweat-rate sensor (Q-sweat, WR Medical Electronics Co.) installed on the positive electrode, the stimulated region is sealed to extract active sweat through an iontophoresis strategy. Through modulating the length of the iontophoresis applied as well as the agonist concentration (e.g., acetylcholine, methacholine, and pilocarpine), the effective sweat-secretion period could be fine-tuned from a few minutes to tens of minutes. In addition, by optimizing the sweat-stimulating concentration of the drug in the custom-developed hydrogels and carefully designing the iontophoresis electrodes, consistent secretion rates exceeding 100 nL/min/cm^2^ could be achieved. Sufficient sweat could be extracted for reliable in situ glucose analysis without causing skin damage or discomfort in the subjects. [12] (Figure 4a). With these merits, iontophoresis is critical in addressing the challenges of increasingly aging populations and diabetic patients with health problems and limited medical services. The composition of exercise sweat is important for applications in athletics and physiology, while sweat generated through chemical iontophoresis is more useful for medical diagnosis. The analysis of such stimulated sweat secretion is significantly more attractive for non-invasive glucose-monitoring applications on account of the short sampling times and the ability to perform sedentary measurements, which is more convenient compared to physical exercise. Iontophoresis may avoid the risks of exercise-induced hypoglycemia in diabetic patients, making it ideal for large-scale population monitoring. Since the body undergoes dynamic physiological changes during exercise and local sweat stimulation through iontophoresis leaves the overall body largely in its resting state, the resulting sweat composition may differ and needs further investigation. However, iontophoresis may be better suited for one-time use rather than continuous monitoring, as the repeated application of iontophoretic current at the same position may be harmful to the underlying skin. As high iontophoresis current can cause skin irritation, all newly developed iontophoretic wearable systems should be designed to reduce the current density and iontophoresis time. The pH of the epidermis will change due to ionic accumulation at the sweat-sampling site due to repeated sweat induction of iontophoresis. Using buffered gel coatings (e.g., cryogel, agarose gel) may aid in preventing burns caused by adverse pH effects, protecting the epidermis, and overcoming this issue [33] (Figure 4b). As the buffered gel inevitably adsorbs into the surface of the epidermis after a while, this incident can raise another safety concern. In short, more research should be focused on the iontophoresis approach to continuous sweat glucose monitoring for effective management of type II diabetes so that it can be used practically and usefully for repeated sweat generation.

#### 3.3.3. Wearable Stretchable Heater for Non-Invasive Continuous Sweat Extraction

Based on its superior electrical conductivity at high aspect ratios, the Ag nanowire network can be used as a highly transparent and stretchable heater for wearable electronics applications [34]. The unique structure of the Ag nanowire network/PDMS as reported by Ko and his co-workers could generate Joule heating with a rapid thermal response, increased electrical stability to withstand repeated mechanical stress (200%), and a small variance in resistance [35] (Figure 4c). For applications requiring non-uniform or site-specific heating, spatial temperature distribution could be controlled by manipulating the spatial current density through the patterning of nanowire percolation networks using direct laser ablation. The soft and thin properties of a wearable stretchable heater allow sweat to be generated on the curvilinear and irregular surfaces of the human body (e.g., chest, forehead, temple, forearm). Long-term sweat generation using wearable stretchable heaters is achieved by coating gold (Au) (a non-toxic, oxidation-resistant material) on the exposed layer of Ag nanowires [34]. The device’s stable, long-term sweat generation for continuous sweat glucose monitoring supports enhanced electrical stability under sweating conditions following Au’s galvanic coating process. However, if the stretchable wearable heater is constructed as a soft, thin, stretchable solid-layer patch, the wearable stretchable heater will be uncomfortable and unbreathable to wear. To overcome this problem, superhydrophobic materials are incorporated to drive sweat into the microfluidic system spontaneously and leave the contact skin area dry and comfortable, even with multiple sweat-generation cycles. This may be a practical technique to generate sweat continuously for non-invasive glucose biosensing, but it needs a power supply. No research has been reported using this approach for sweat glucose monitoring in situ.

#### 3.3.4. The Collection and Detection of Sweat Components at Rest via Hydrogel

The majority of wearable sweat sensors have focused on exercise, chemically (iontophoresis), or thermally driven sweat production, but they are unable to swiftly draw tiny volumes of resting sweat into the device, restricting real-time analysis. In the past, bulky instrumentation or 24 h collection periods for single-point analyte measurement were necessary for at-rest thermoregulatory sweat-rate monitoring in clinical settings [36]. There is still a need for convenient, wearable technologies for continuous resting-sweat monitoring. This is a major hurdle that must be addressed for sweat to become a viable means of health monitoring across all activities, whether active or sedentary, and across all user groups, whether young or elderly, healthy or sick. Recently, Nyein et al. (2021) developed a wearable hydrogel patch for continuous analysis of thermoregulatory sweat at rest for pH, Cl^−^, and levodopa monitoring [37]. The microfluidic device was designed to enable effective small-volume collection and analysis of resting sweat. The researchers demonstrated a thin hydrogel patch functionality for dynamic sweat analysis in the context of everyday activities, stressful events, hypoglycemia-induced sweating, and Parkinson’s disease.

In another study by Sempionatto et al. (2021), a new rapid and reliable approach that combines a simple hydrogel touch-based fingertip sweat electrochemical sensor with a new algorithm that addresses personal variations toward the accurate estimate of blood glucose concentrations was presented [38]. This new painless and simple glucose self-testing protocol leverages the fast sweat rate on the fingertip for rapid assays of natural perspiration without any sweat stimulation, along with the personalized sweat-response-to-blood concentration translation. In another earlier study, Nagamine et al. (2019) also reported on a hydrogel-based touch-sensor pad for the non-invasive extraction and detection of sweat components [39]. The sensor was composed of electrochemical biosensing working and reference electrodes fully covered with an agarose hydrogel, including a sweat-extraction solution, Dulbecco’s Phosphate Buffer Saline (DPBS). The sweat components can be easily and continuously extracted from human skin contact with the agarose hydrogel at rest, followed by in situ L-lactate detection. In another study that utilized the fingertip as a site for simultaneous biomarker data sampling and user identification, Prof. Emaminejad (2020) reported the use of a thin hydrogel micropatch that exploits sweat to noninvasively access biomarker information at rest [40]. The fingertip was determined as the optimal site for natural perspiration sampling, which was subsequently used to demonstrate the successful tracking of the dosage level and metabolic pattern of caffeine intake among different subjects.

All the above-reported studies utilized hydrogel to collect the resting sweat. However, the main issue is that utilizing hydrogel alone as a sweat-extracting platform at rest can dilute sweat compositions and hence put a challenge on the detection limit and sensitivity of electrochemical sensors. Instead of using only a thin hydrogel layer, the researchers elegantly occupied the remaining dead space with the rigid filler. Eventually, these hydrogel patches offer continuous, autonomous monitoring of body physiology at rest by enabling sweat analysis suitable for sedentary, habitual, and daily activities.

### 3.4. Wearable Self-Powered System

Currently, most wearable batteries are rigid, substantially bulky, and require external charging or regular replacement. In addition, there are intrinsic safety concerns with commonly used batteries (e.g., lithium batteries) [41]. The electrochemical biosensing system should therefore perhaps be combined with a self-powered energy harvesting, conversion, and storage system for routine continuous sweat glucose monitoring.

#### 3.4.1. A Wearable Solar Cell and Biofuel Cell

Clean and renewable energy from the surrounding environment (e.g., solar energy, mechanical movements and frictions, biofluids, etc.) could be used to create a self-powered system to support the effective operation of wearable glucose biosensors. Compared to flammable, organic electrolyte-based batteries, the self-powered solar energy system eliminates safety concerns at higher cell temperatures, particularly with relatively large outdoor heat generation. Zhao et al. (2019) reported an integrated self-powered wearable glucose biosensor that can be charged up to 6.0 V in 1 h of outdoor sunlight (approximately 1.5 air mass) [25]. The unit reached a cruising time of up to 8 h with a cut-off potential of 3 V with the solar energy contained in the batteries. Nevertheless, for everyday use in continuous sweat glucose monitoring, a 1 h charge in outdoor sunlight may not be practical. Biofuel cells, on the other hand, could be an alternative solution to the problem. Nevertheless, most of the wearable biofuel cells produced are based on epidermal platforms that use sweat lactate as biofuel [28]. This is rational since lactate and glucose are excreted from the sweat gland at the same time, and their concentration depends on the individual based on their eating habits [42]. A stable supply of biofuel is essential for the efficient production of electricity. This problem can be overcome by combining a powerful sweat generator with a sweat-rate sensor and microfluidics to ensure that effective sweat flows continuously. On the other hand, by designing biocatalysts and enzyme–electronic interfaces with lower resistance, the main stability problem can be mitigated. Finally, it is possible to improve power generation by integrating enzymatic cascade reactions and also connecting the biofuel cell systems themselves with biocomputing logic-gate systems for reversible on/off switching of power output [43].

#### 3.4.2. A Wireless Power Transmitter

In addition to fuel cells, WiTricity (wireless electricity) could provide a powerful solution to the wearable electrochemical biosensor energy supply problem [44]. WiTricity is a new advanced technology that demonstrates that wireless energy can be delivered to a wearable biosensor over a moderate distance to monitor sweat glucose autonomously and in real time. The WiTricity theory is based on magnetic resonance (coupled-mode theory), strongly coupled. First, compact WiTricity resonators are designed, followed by the construction and evaluation of a working prototype of a WiTricity-driven wearable biosensor. An energy transfer efficiency of around 80% over a distance of 15 cm will allow the integrated system to function properly. The wearable sweat glucose biosensor can be powered wirelessly by inductive transmitter coupling antennas. A certain misalignment between the transmitter and the receiver will have little effect on the power transfer and will affect the efficiency of the power transmission. Lateral and angular misalignments of the receiver antennas could be solved using a configuration of multi-transmitter antennas. With the use of in-phase transmitter antennas, the active range over which the wearable biosensor could function properly could be enhanced. Wearable biosensors can work at a range of 4–10 cm continuously. The wireless power-management system could reduce the wearable glucose biosensor’s weight, eliminate safety problems, and enable continuous monitoring of sweat glucose over extended periods.

#### 3.4.3. A Small Flexible/Wearable Aqueous Rechargeable Battery

For a good self-sustaining system, attention should be paid not only to energy harvesting and conversion but also to power storage. The latest advances in research on stretchable aqueous batteries, especially aqueous Li-ion batteries and zinc-based batteries, are important for the realization of wearable devices of the next decade, such as sensors, medical devices, and electronic skin [45]. An aqueous rechargeable zinc-manganese oxide (Zn-MnO_2_) battery with unique features such as improved safety, lightweight, eco-friendliness, high output voltage, and high capacity has become one of the most promising alternatives to replacing conventional lithium batteries or any commercially available batteries using flammable organic electrolytes [25]. The Zn-MnO_2_ batteries could be used as intermediate energy storage systems, and the use of aqueous electrolytes could reduce battery safety issues, which are critical for wearable devices. Such remarkable energy-storage capacity and mechanical strength could provide versatile and portable applications for the fabricated flexible battery.

### 3.5. Flexible, Microfluidic Sweat-Sampling System

**Figure 5:**
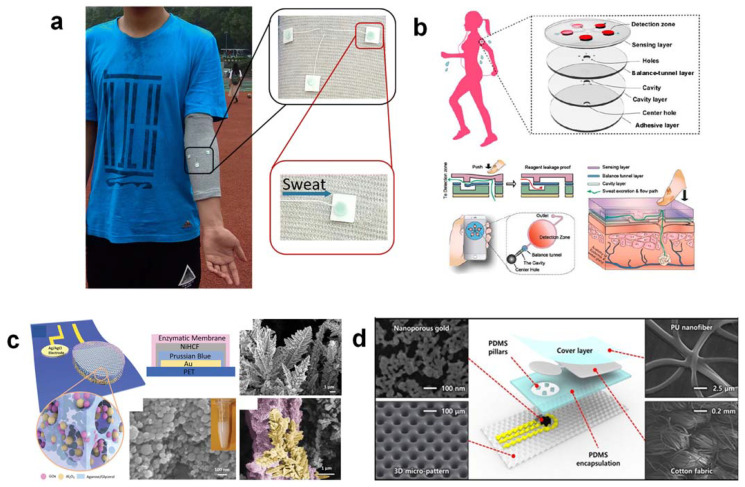
(**a**) A wearable microfluidic cotton thread/paper-based device linked to a smartphone for sweat glucose sensing. (**b**) A wearable colorimetric sensor based on microfluidic chips that detect sweat glucose easily and quickly. (**c**) Porous enzymatic membrane for highly stable nanotextured glucose sweat sensors to ensure reliable, non-invasive health monitoring. (**d**) A nanoporous electrochemical gold capillary microfluidic integrated sensor that is fully stretchable for continuous glucose monitoring. The figures are reprinted with permission from ref [8,49,50].

For a wearable sweat glucose biosensor without integration with a microfluidic system, there are four main challenges. The challenges are (1) susceptibility to skin (bio)marker contamination; (2) mixing and carryover between new and old sweat; (3) irreproducible sample transport over the surface of the detector; and (4) lack of sweat evaporation and volume control. As a result, for sweat collection, capture, storage, and analysis, a small, flexible, skin-compatible microfluidic device with microfluidic channel networks, inlet/outlet ports, micro-reservoirs, and electrochemical/colorimetric biosensors is required [46]. In the past, fabric (cotton threads) was used as a medium for the manufacture of low-cost and low-volume microfluidic devices [8] (Figure 5a). Nowadays, advanced strategies such as laser ablation enable quick material patterning on small scales and are useful for the gravure of microfluidic channels in flexible plastic substrates [47]. The integration of microfluidics into wearable sweat glucose biosensors overcomes many challenges that decrease data integrity. First, when sweat reaches the microfluidic channels, it is separated from the surface of the skin, stopping the skin from constantly leaking chemicals into the sweat. To reduce the effects of mixing and carryover, efficient sweat sampling often requires cleaning the area of the skin on which the sweat is collected, as residual glucose and exogenous contaminants can affect the measurement. Second, channels can be built so that sweat can be continuously replenished by steering old sweat away from the biosensor and allowing new sweat to flow in. This guarantees readings as close to real time as possible rather than generating rolling averages of sweat glucose concentrations due to sweat-dilution problems caused by varying sweat rates. Third, microfluidics enables consistent measurements at low sweat rates. Fourth, microfluidics can encase sweat while traveling through the biosensing electrodes, minimizing evaporation, preventing environmental or external contamination, and avoiding direct contact with the human skin of the sensing components [4]. In addition, the microfluidic channel connected to the electrochemical biosensors should be made moderately hydrophobic by short-term heat treatment before bonding to the electrochemical sensors. This is to prevent any calibration solution from flowing back to the microfluidic channel or contaminating it. Xiao et al. (2019) have recently developed a wearable colorimetric sensor based on soft, microfluidic chips that can be placed directly on the skin to detect sweat glucose [48] (Figure 5b). The device consisted of five microfluidic channels linked to the detection of microchambers. Multiple sweat controls and inlets are necessary because they are intended for efficient sweat collection. The microchannels redirected the sweat excreted from the epidermis to the microchambers, and each of them was combined with a control valve to avoid the risk of microchamber backflow of chemical reagents. In short, microfluidics with multiple sweat-uptake channels and a safety check valve are the most important criteria for designing a functional system, especially for in situ sweat glucose colorimetric biosensing.

Soft microfluidic devices with shorter sweat sampling, filling times, and advanced detection methods are needed to provide timely and rich analytical data. Nonetheless, this must be theoretically modeled and experimentally implemented to achieve short sweat-sampling times, quick sweat flow rates, and effective transportation over the detector surface [17]. The increased sweat-sampling level could effectively address several existing challenges of accurate epidermal electrochemical glucose biosensing by continuously providing sufficient sweat to the detector surface for robust biosensing while rapidly removing initial contaminating glucose residues on the skin surface or in sweat pores. Nevertheless, due to the proximity of openings, a greater number of inlets would facilitate mechanical instability of the adhesive sheet, which could lead to sweat leakage. As a result, the maximum number of inlet configurations that allow rapid sweat sampling without significantly increasing sweat volume (small sweat volumes at the microliter level) should be carefully engineered, resulting in a short surface-detector filling time. The conditions between tests should be preserved since temperature and sweat-extraction conditions will change the sweat rate and therefore the filling time of the detector surface.

### 3.6. A Porous Interface to Enhance Sweat Glucose Biosensing Sensitivity

Porous electrodes (e.g., Al_2_O_3_, MXene) could enhance the loading of GOx enzymes used for glucose biosensing and exhibit a higher sensitivity than planar electrodes, as porous electrodes usually have a larger electrochemically active surface available for electrochemical reaction with reactants, particularly with a small amount of sweat glucose [18]. In addition, porous electrodes can reduce potential drifts at redox peaks due to differences in the concentration of electrolytes and charging effects compared to planar electrodes [49] (Figure 5c). Robustly cross-linked enzymes on the porous metal structure enable stronger enzyme immobilization, prevent delamination of the enzyme membranes, and improve the biosensor’s reliability under mechanical friction and deformation [50] (Figure 5d).

### 3.7. High-Throughput Roll-To-Roll (R2R) Device Fabrication Technique for Large Population Studies

**Figure 6:**
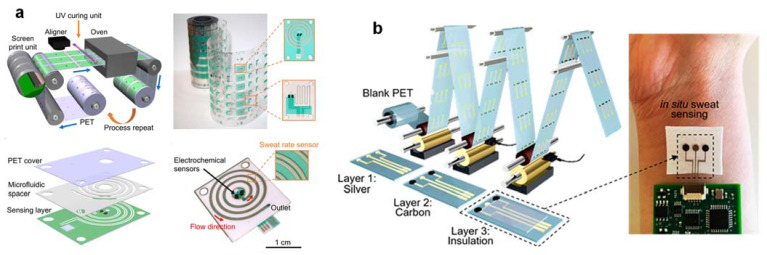
(**a**) Regional and correlative sweat analysis using microfluidic high-throughput sensing patches to decode sweat. (**b**) Roll-to-roll electrochemical sensors for wearable and medical devices. The figures are reprinted with permission from ref [51,52].

One of the main obstacles to large-scale population studies needed to interpret sweat is the achievement of high-throughput sweat glucose biosensors with uniform, reliable performance (with high stability) and high yields that are also necessary for commercial viability. Roll-to-roll (R2R) rotary screen printing is a promising method for producing high-performance and cost-effective flexible wearable electronics [51] (Figure 6a). These R2R-compatible methods are advantageous over traditional, multi-stage lithography and etching processes since components can be mass-produced at high speeds on large substrates using automated systems with minimal human involvement [52] (Figure 6b). The R2R fabrication of integrated sweat-rate sensors and microfluidics is a crucial application-oriented technological advancement to help decode the sweat glucose-secretion rate-dependent modulation. To date, the iontophoretic sweat glucose dynamics and sweat-to-blood glucose correlations have not been adequately studied for the feasibility of sweat-based diabetes management. When considering iontophoresis sweat glucose and sweat rate alone with simple models, it would be difficult to predict instant blood glucose levels accurately. This does not preclude the possibility of a more complex relationship between sweat components and blood glucose, including multiparameter and lag time dependence [51]. Expanding basic knowledge of sweat gland physiology and carrying out large-scale correlation studies with multiplexed sensing to characterize sweat-rate dependencies could help unravel the secret. In short, the design of robust and flexible R2R gravure printed electrodes is a crucial translation step in making it possible to produce large-scale, low-cost wearable glucose biosensors for personalized health-monitoring applications.

### 3.8. Correlation with Blood Glucose

Concentrations of sweat glucose have a time lag, and the concentration range varies from that of blood glucose due to diffusion barriers in human physiology. A physiological basis for knowing the sweat glucose-secretion process could provide a physiological basis for more sophisticated models that account for or normalize sweat rates, leading to a stronger relationship with blood glucose [53]. While the lack of a uniform sweat-to-blood glucose correlation across all subjects makes it difficult to identify universal sweat thresholds for diagnosis or management of type II diabetes, more thorough longitudinal sweat glucose studies should result in individual-specific sweat-to-blood relationships for personalized diabetes management because the correlation for each individual is different. Standard techniques such as Clarke Error Grid Analysis, used for every new device’s quality evaluation for human blood or plasma glucose detection, should also be used for human sweat glucose detection [54]. Big-data analytics should be the basis of precision and proactive medicine. For effective management of type II diabetes lifestyle disease, advanced big-data analytics and Artificial Intelligence (AI)-based diagnostic and predictive tools (e.g., Machine Learning (ML) or Deep Learning (DL)) to elucidate the correlation between sweat and blood glucose would be useful [55]. Nevertheless, the most important consideration of basal sweat and blood glucose changes is sweat assessment to diagnose prediabetes or diabetes and to predict the initiation of glucose events (e.g., hypoglycemia) to commence early and effective treatment.

### 3.9. Glucose-Triggered Insulin and Therapeutic Drug Delivery Close-Loop System for Precision Theranostics

**Figure 7:**
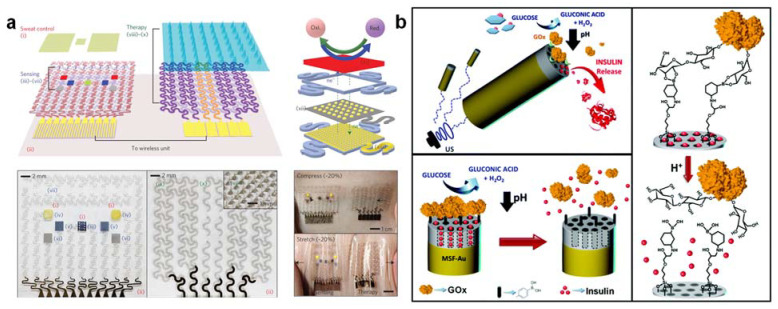
(**a**) A graphene-based electrochemical device with thermoresponsive microneedles for diabetes monitoring and treatment. For explanation for (i)–(vi) in subfigure a, please refer to Figure 1, ref [57]. (**b**) Biomedical nanomotors for efficient glucose-induced insulin release. The figures are reprinted with permission from ref [56,57].

Maintaining a normal glucose level is critical to health management, as high and low glucose levels may indicate a risk of metabolic disorders such as diabetes or hypoglycemic shock, especially after intense exercise or an overdose of glycemic control drugs (e.g., metformin, aspirin, chlorpropamide, Tylenol). The current medical treatment of diabetes patients is focused on the self-monitoring of blood glucose concentration and the subcutaneous self-administration of insulin to maintain normal glycemic levels. This common treatment procedure, however, is intrusive, stressful, and painful, and thus subject to insufficient glycemic control, which can lead to additional complications in health. Significant efforts have been devoted to the design of “closed-loop” therapeutic systems capable of supplying insulin [56] or metformin [57] in response to increased levels of sweat glucose. A fully integrated device for high-fidelity sweat glucose measurement and feedback-controlled insulin or drug delivery enables effective, pain-free management of blood glucose concentration. Ideally, this closed-loop therapeutic unit can inject insulin/metformin at hyperglycemic peaks and glucagon at hypoglycemic pits for effective blood glucose control. The closed-loop therapeutic system could be either a device for transdermal drug delivery (e.g., the temperature-responsive phase change of nanoparticles embedded with microneedles) [57] (Figure 7a) or pH-sensitive nanocarriers [56] (Figure 7b). Since drug delivery through the skin can bypass the digestive system, insulin and metformin transdermal delivery requires a lower dose of drugs than oral delivery and thus prevents gastrointestinal side effects [58]. The integrated wearable tremor sensor detects simulated tremors under low blood glucose conditions that could be induced in hypoglycemic states to inject glucagon. In addition to the temperature-responsive phase-change nanoparticles, particular attention was also paid to glucose-responsive materials based on GOx’s biocatalytic reaction [59,60]. GOx catalyzes glucose conversion into gluconic acid with a decrease in local pH. For the development of stimulus-responsive therapeutic-agent-delivery systems, pH-sensitive nanocarriers based on polymeric nanogels, nanocapsules, and mesoporous silica could be combined with GOx [61]. Nevertheless, the development of wearable bio-responsive nanomachines integrating continuous movement and glucose biosensing with activated insulin/drug release for theranostic precision remains a medical challenge unmet.

### 3.10. Security and Privacy Issues for Personalized Medicine in Wireless Wearable Biosensor Networks

Since most wearable devices and their data and signal transmission to an external mobile device or gadget are wireless in nature, security and privacy are major concerns [62]. The sensitivity also increases due to the direct involvement of humans. Whether the data collected from patients or individuals are obtained with or without the consent of the subjects, due to system requirements, misuse, or privacy concerns, may restrict end-users from taking full advantage of the integrated and autonomous wearable glucose biosensors. Furthermore, although the main intention is noble, some end-users may not consider such devices safe for everyday use. In the worst scenario, serious social concerns may also arise due to the fear that such devices may be used by government agencies or other private organizations to monitor and track individuals. Early detection of such a breach would reduce safety risks and ensure the privacy, accuracy, and integrity of health care data generated by wearable sweat glucose biosensors.

## 4. Conclusions and Future Perspectives

In the field of predictive diagnosis and routine monitoring of diabetes mellitus, it is crucial to develop wearable glucose biosensors with superior reproducibility and sensing stability for accurate, non-invasive glucose monitoring. Robust and stable components of biosensing need to be developed to enable long-term use. This is because the long-term stability and uniformity of biosensors make the system applicable to humans with minimum recalibrations. The incorporation of microfluidics and multiplexed sensing to improve data integrity must be combined with a higher-level study of the physiological relevance of sweat glucose analysis using in situ large-scale correlation studies. Theranostic interventions with autonomous closed-loop insulin or therapeutic-drug delivery systems could improve glycemic control. Parallel efforts are also needed to ensure secure data-handling and to adopt high-throughput manufacturing methods so that wearable sweat glucose biosensors can be used more widely. The physiological importance of sweat glucose has yet to be investigated to assess its utility in realistic type II diabetes control and management applications. As for prospects, apart from roll-to-roll printing, the use of 3D printing to produce a wearable sweat glucose biosensor will solve reproducibility and long-term stability problems. Three-dimensional printing enables precise and intricate designs to be produced on small scales that are useful for ultra-low sweat volumes. Three-dimensional-printed glucose biosensors can perform better when gathering glucose signals by incorporating pH, temperature, and sweat-rate sensors, making them better than conventional electrode methods, and the produced biosensors can be more flexible as a personalized medicine to meet a variety of end-user biological needs. By combining 3D-printed biosensors with electronic components on wearable devices, large-scale use would be possible. The manufacturers could use the same 3D printer nozzles used to print the glucose biosensors to print other components (e.g., microfluidic, iontophoresis, stretchable metal nanowire heaters, PCB) of the wearable devices to reduce the risk of manufacturing defects in the assembly and manufacture of fully integrated devices. Wearable sweat glucose monitoring systems could be incorporated using the Internet of Things (IoT) to improve the quality of services for diabetes care. The feasibility of an IoT-based approach for non-invasive and continuous glucose monitoring could be further explored. An IoT-based system architecture could be built from a wearable sweat glucose biosensor unit to a back-end system for displaying real-time sweat glucose, skin temperature, local pH, contextual information (i.e., sweat rate), and blood glucose correlation in graphical and human-readable forms for end-users (patients and doctors). On the other hand, the RF communication protocol could be tailored to suit the sweat glucose monitoring system, allowing a high energy efficiency level. The IoT-based system provides many advanced gateway-level services, such as a smart theranostic system by triggering insulin or therapeutic drug injections via the epidermis in abnormal situations (i.e., hypoglycemic or hyperglycemic). The ability to simultaneously measure both glucose and insulin would enhance glucose regulation and better management of type II diabetes by providing an improved insulin tolerance estimate, reducing medication variability, and optimizing hypoglycemic protection. Insulin, however, cannot be present in sweat, but it can be measured in saliva [63] and tears [64]. Direct detection of free insulin by wearable affinity biosensors, either in saliva or tears, should be able to overcome the challenges posed by the size of the molecule, the low insulin concentration, and the selectivity against interferers in these biological mediums. In short, efficient, realistic, and continuous monitoring of sweat glucose and saliva/tear insulin using a wearable non-invasive point-of-care system offers significant potential for reducing the morbidity and mortality rate caused by diabetes mellitus and preventing epidemic diabetes.

## Data Availability

Not applicable.
